# Learning from the past: Intergenerational transmission of aggressive conflict resolution between intimate partners predicts harsh and inconsistent parenting

**DOI:** 10.1111/jora.70102

**Published:** 2025-11-30

**Authors:** Pin Chen, Sanne B. Geeraerts, Susan Branje

**Affiliations:** ^1^ Department of Experimental Psychology University of Oxford Oxford UK; ^2^ Youth and Family, Department of Education and Pedagogy Utrecht University Utrecht The Netherlands

**Keywords:** conflict resolution, discipline, gender, intergenerational transmission, spillover effect

## Abstract

The study examined the intergenerational transmission of aggressive conflict resolution toward intimate partners from Generation 1 (G1) parents during Generation 2's adolescence to both G2 and their partners (G2 partner), and the potential spillover effects from G2 and G2 partner's aggressive conflict resolution to G2's harsh and inconsistent parental discipline towards Generation 3 (G3) children. Using data from the Research on Adolescent Development and Relationships (RADAR) project, G1, G2 (51.5% girls; *M*_*age*_ = 14.82 in mid‐adolescence, *M*_*age*_ = 29.66 in parenthood), and later G2's partner and G3 were followed from G2's adolescence to adulthood. The sample comprised 1178 G1–G2 dyads, including 222 G1–G2–G3 triads. Path analyses provided evidence for (1) intergenerational transmission, that is, G1's aggressive conflict resolution in G2's mid‐adolescence weakly predicted G2's aggressive conflict resolution in G2's adulthood and (2) spillover effects, that is, G2's aggressive conflict resolution predicted G2's harsh and inconsistent discipline toward G3 children. Most of the intergenerational transmission and spillover relations did not differ across G1 and G2 gender. Overall, the findings highlight the intergenerational transmission of aggressive conflict resolution towards intimate partners and its subsequent relation with harsh and inconsistent discipline. Future prevention could target both parental figures in G1 and G2 to disrupt the cycle of aggressive conflict resolution and prevent problematic discipline practices.

## INTRODUCTION

As adolescence is a period in which youth gain perspective‐taking skills (Van der Graaff et al., 2014) and their ability to regulate their emotions increases (Maciejewski et al., 2019), it is key for learning and experimenting with conflict management (Laursen et al., 2001). Conflict resolution learned from parents during this stage can have effects on individuals' behaviors in family, peer, and romantic relationships (Staats et al., 2018; Van Doorn et al., 2007). This study examined whether parents' aggressive conflict resolution during their child's adolescence shapes the child's behavior in young adulthood—especially in their intimate partnerships and parenting practices. We investigated the intergenerational transmission of aggressive conflict resolution from first‐generation (G1) parents to their second‐generation (G2) children's romantic relationships, and how G2's aggressive conflict resolution relates to their harsh and inconsistent parenting towards their offspring (G3).

### Intergenerational transmission of aggressive conflict resolution

Aggressive conflict resolution—characterized by physical or verbal aggression, such as personal attacks during disputes (Kurdek, 1994)—may be transmitted across generations. The Dynamic Developmental Systems (DDS) model provides an integrative framework to understand this transmission, positing that both individual predispositions (e.g., genetics) and contextual influences (e.g., social learning) contribute to intergenerational transmission (Kerr & Capaldi, 2019). Among nongenetic mechanisms, social learning theories offer a compelling explanation (Bandura, 1977; Ehrensaft & Langhinrichsen‐Rohling, 2022). When children witness aggressive conflict resolution between parents, they may perceive it as normative, internalize these behaviors, and later replicate them in their own romantic conflicts (Kerr & Capaldi, 2019; Ribes‐Inesta & Bandura, 1976). Thus, these theories suggest a potential link between aggressive conflict behaviors of parents and those of their children.

Intergenerational transmission of aggressive conflict resolution may also manifest in adolescents' romantic partners through social learning. Adolescents exposed to parental aggressive conflicts may view such behavior as acceptable (Cui et al., 2010), increasing the likelihood of selecting partners who also use aggressive conflict strategies. Although parents' conflict behavior may not directly influence adolescents' partners, a positive association is expected between parents' and partners' use of aggressive conflict resolution.

Conflict is inevitable in close relationships, making the strategies used to resolve it particularly important. Aggressive conflict resolution is typically lower in intensity but more frequent than overt aggression in everyday life and has been linked to poorer psychological adjustment and strained relationships in both adolescence (Branje et al., 2009) and adulthood (Schudlich et al., 2013). While prior research has examined the intergenerational transmission of relational aggression across two or three generations (e.g., Cui et al., 2010; Doumas et al., 1994), they might have assessed aggression outside of conflict contexts, potentially reflecting motives such as dominance or emotional release rather than dispute resolution. Surprisingly little is known about the transmission of aggressive conflict resolution specifically—despite its likely higher prevalence. Two prospective studies have found intergenerational transmission of conflict intensity from individuals' adolescence to adulthood (Hsieh et al., 2022; Rothenberg et al., 2016), but these conflicts involved various family members, not just romantic partners. Additionally, many studies of romantic conflict failed to distinguish between adolescents' and their partners' behaviors, conflating actor and partner effects. Disaggregating these roles is essential to clarify whether the intergenerational transmission is from the parents to the young adult child or to their partners. For example, if adolescents tend to choose partners who use aggressive conflict resolution rather than engage in such conflict resolution themselves, interventions might focus on enhancing partner selection skills and supporting their partners' conflict management.

### Role of adolescent and parent gender in intergenerational transmission

The intergenerational transmission of aggressive conflict resolution may differ by adolescent gender, but findings are mixed. Some studies reported a transmission of family conflict intensity (Rothenberg et al., 2016) and intimate partner violence (Smith et al., 2011) from family of origin during adolescence to later intimate partnerships in adulthood only in girls, who are often socialized to caretake family relationships and may be more strongly affected by interparental conflicts (Buehler & Gerard, 2002). Conversely, a meta‐analysis found stronger effects of exposure to domestic violence on externalizing behavior during adolescence for boys (Evans et al., 2008), in line with theories that patriarchal norms may normalize men's use and women's experience of aggressive behavior (Reese‐Weber & Kahn, 2005). Other studies found no gender differences in effects of interparental aggression during adolescence on later aggression in romantic relationships (Cui et al., 2010) and in effects of interparental conflict on aggressive behavior in adolescence (Li et al., 2023). Despite extensive work on general aggression and family conflict, it remains unclear whether the intergenerational transmission of aggressive conflict resolution differs by adolescent gender. Furthermore, few studies have explored the long‐term effects of interparental conflict resolution during adolescence on a child's future conflict behaviors in young adulthood.

Parent gender may also play a role in the transmission. The gender‐specific hypothesis, grounded in social learning theory (Bandura, 1977), posits that children are more likely to model same‐gender parents, as these models demonstrate gender‐appropriate behaviors that are more often rewarded (Kwong et al., 2003; Perry & Bussey, 1979). However, empirical support for gender‐specific transmission in aggressive conflict resolution is limited and mixed. Some evidence supports same‐gender transmission in related behaviors like criminality (Auty et al., 2017), while studies on spouse abuse (Kalmuss, 1984; Kwong et al., 2003; Smith et al., 2011) and antisocial behavior (Tzoumakis et al., 2020) do not. Cross‐gender effects have also been observed, for example, stronger transmission from fathers to daughters in externalizing behaviors (Kim, Capaldi, et al., 2009) and intimate partner violence perpetration (Shakoor et al., 2022).

Given these inconsistencies, the current study examined (1) moderation by adolescent gender and (2) parental‐by‐adolescent gender interactions (i.e., gender‐specific pathways). By exploring these gender dynamics, we aimed to add evidence to the literature and identify potential at‐risk groups for targeted prevention based on the gender in both generations.

### Spillover effects of aggressive conflict resolution to harsh and inconsistent discipline

Aggressive conflict resolution used by individuals and their partners may spill over to individuals' use of harsh and inconsistent discipline, characterized by coercive, aggressive, and contrasting measures to control child behavior (Gardner, 1989; Verhoeven et al., 2017). Parents who resolve conflicts aggressively may view coercion as a viable approach to handle disputes (Bandura, 1973) and manage misbehavior. Frequent aggressive conflict in romantic relationships can also lead to emotional exhaustion, reducing the capacity for consistent (Kaczynski et al., 2006; Warmuth et al., 2020) and effective parenting (Van Dijk et al., 2020).

Although prior studies have supported spillover effects of destructive interparental conflict on harsh and inconsistent discipline (Buehler & Gerard, 2002; McCoy et al., 2013; Neppl et al., 2019; Sears et al., 2016; Warmuth et al., 2020), they often treat conflict as a general construct without differentiating who displayed which conflict behavior. However, as different mechanisms may underlie young adults' and partners' conflict behavior, this study separately examined conflict behaviors from young adults and their partners to test their distinct spillover effects on young adults' parenting.

### Role of gender in spillover

The spillover of aggressive conflict resolution into harsh and inconsistent parenting may vary by young adults' gender. Some research suggested that men are more vulnerable than women to the adverse impact of relational conflict on parenting (Belsky et al., 1991; Brody et al., 1996; Sturge‐Apple et al., 2004). Social Role Theory (Thompson & Walker, 1989) offers a possible explanation: men may have less clearly defined parenting roles than women, making it more challenging for them to separate and compartmentalize their roles as partners and parents. Hence, marital conflict may more strongly affect men's parenting behavior (Belsky et al., 1991). This gender difference has been supported by multi‐method studies using observations and physiological data (e.g., Levenson & Gottman, 1983; McCoy et al., 2013; Stroud et al., 2011). However, other studies suggest the opposite—women high in aggression may struggle more with caretaking roles (Elder et al., 1986; Rothenberg et al., 2016) and be more likely to engage in harsh and inconsistent discipline. Despite these mixed findings, most prior research has combined men's and women's conflict behavior into a single variable, overlooking potential gender differences in spillover processes (Kopystynska et al., 2017). To address this gap, the current study separately examined young adults' and their partners' aggressive conflict resolution and its association with adolescents' parenting, allowing us to identify potential gender‐specific patterns of spillover.

### Linking three generations: intergenerational transmission and spillover

Combining the concepts of intergenerational transmission and spillover, there may be a three‐generational pathway from parents' aggressive conflict resolution during an adolescent's upbringing to that adolescent's own parenting behavior in young adulthood. This three‐generational association may be mediated by the adolescents' and their partners' use of aggressive conflict resolution in young adulthood.

Studying conflict and parenting from a three‐generational perspective is important for understanding the long‐term influence of interparental conflict in the family of origin on both romantic relationships and parenting in the next generation. Prior research has shown that individuals who experienced household dysfunction in childhood—such as interparental aggression—were more likely to engage in harsh or negative discipline as parents (Lotto et al., 2023; Rowell & Neal‐Barnett, 2022). Longitudinal studies like the Family Transitions Project (Martin & Conger, 2018) have documented the transmission of aggression across three generations and the spillover of conflict behaviors from partnerships to parenting, measured from adolescence to adulthood. For instance, Conger et al. (2003) showed that antisocial and aggressive behaviors can be transmitted across three generations, tracking individuals from adolescence to parenthood of their 2‐year‐old children. Furthermore, Neppl et al. (2019) found that intimate partner violence spilled over to harsh parenting. In the same work, Neppl et al. (2019) also found that parental violence toward individuals during adolescence was associated with individuals' harsh parenting through their violence perpetration toward partners. Based on these findings, the current study examined whether interparental aggressive conflict resolution predicted adolescents' later use of harsh and inconsistent parenting, and whether this association was mediated by both adolescents' and their partners' aggressive conflict resolution in young adulthood, a potential leverage point for disrupting the cycle of aggressive conflict resolution across generations.

### Current study

Using prospective, longitudinal data, the current study aimed to provide a rigorous and nuanced understanding of how aggressive conflict resolution patterns are transmitted and maintained across generations. We examined whether interparental conflict resolution during participants' adolescence was associated with their own aggressive conflict resolution in young adulthood, and whether this was subsequently associated with their parenting behavior. We differentiated between the individuals enacting and experiencing aggressive conflict resolution—both within the parent generation (i.e., mothers and fathers) and the adolescent generation (i.e., adolescents and their partners)—to clarify whether these patterns differed by gender. We examined four main hypotheses: First, parents' use of aggressive conflict resolution with intimate partners in their child adolescents' mid‐adolescence would be positively associated with both adolescents' and their partner's aggressive conflict resolution in adulthood. Second, adolescents' and their partners' aggressive conflict resolution during young adulthood would be positively associated with adolescents' harsh and inconsistent discipline practices as parents. Third, adolescents' and partners' aggressive conflict resolution during young adulthood would be positively associated with each other, given the interactive nature of conflict behavior within romantic relationships (Ehrensaft & Langhinrichsen‐Rohling, 2022). Fourth, parents' aggressive conflict resolution would be positively associated with adolescents' harsh and inconsistent parenting during young adulthood, mediated by the aggressive conflict resolution used by adolescents and their partners. Finally, we explored the role of gender in these pathways. Based on the gender‐specific hypothesis (Kwong et al., 2003; Perry & Bussey, 1979), we expected stronger transmission in same‐gender parent–child dyads than in mixed‐gender dyads. We also tested whether adolescents' gender moderated the intergenerational transmission and spillover effects, given prior mixed evidence on gender differences in conflict and parenting outcomes.

## METHODS

### Participants and procedure

The current study used data from the ongoing longitudinal study, The Research on Adolescent Development and Relationships (RADAR; Branje & Meeus, 2018; Van Lier et al., 2011). Ethical approval was initially obtained from the University of Medical Centre Utrecht and for later waves by the Ethics Review Board of the Faculty of Social and Behavioral Sciences of Utrecht University (FETC24‐0157). Two cohorts of participants began participating in their first year of high school in 2001 and 2005, respectively. Participants (Generation 2; G2), their parents (G1), and later intimate partners, have been tracked annually and later biennially to participants' adulthood. G2 participants were invited for data collection in Generation 3 (RADAR‐G3) once they had children (G3). All participants provided active consent.

The participants' ages varied within each wave. Specifically, the wave in which G2 participants were in their mid‐adolescence (14–17 years old) was labeled as T1 and the wave in which G3 children were 2 years old was labeled as T3. The last wave in which G2 participated prior to T3 was labeled as T2. To further maximize data utilization, we included G2 participants who did not have children and therefore lacked data at T3. For these participants, we calculated the modal interval of waves between T1 and T2 in the data set of participants with G3 children (i.e., eight waves) and used this interval to assign a T2 wave for participants without children participating. These participants, by design, had available data only at T1 and T2. Data collection and rationales for participant inclusion are detailed in the Supplement S1. Participant eligibility and inclusion among G1‐G2 dyads and G1‐G2‐G3 triads are presented in the Supplement S8 (Figure S1).

**FIGURE 1:**
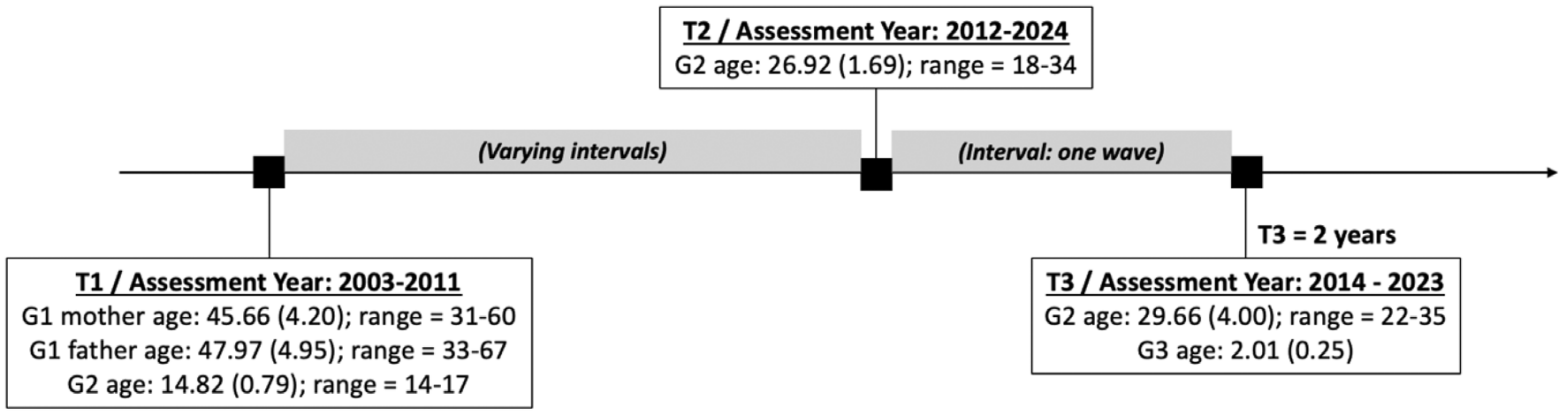
Mean and standard deviation of participant age throughout the study. Values in parentheses indicate the standard deviations of age. The one‐wave interval between T2 and T3 applies only to G2 participants with participating G3 children.

The current study comprised 1178 participants with data on the G1–G2 measures, among which 222 participants had data on G1–G2–G3 measures. Among all G2 participants (*N* = 1178), 621 of them had partnerships at T2. For the biological sex of all G2 participants, used as a moderator in analyses, 51.5% were girls. The mean age of all G2 participants was 14.82 (*SD* = 0.79; range = 14–17 years) at T1, 26.92 (*SD* = 1.69; range = 18–34 years) at T2, and 29.66 (*SD* = 4.00; range = 22–35 years) at T3. All G2 participants self‐identified as Dutch. At T2, 53.7% (G2 with G3 children: 94.6%) lived with their partners, and 79% reported a monthly household income at or above the national average in 2022 (2375–4267 euros, Centraal Bureau voor de Statistiek, n.d.). Among G2 participants who reported gender (*n* = 808), 379 (46.9%) identified as men, 427 (52.8%) as women, 1 as a transgender man, and 1 as gender queer (not exclusively male or female). Of G2 partners who reported personal information, 93.6% (*n* = 560) self‐identified as Dutch; among those reporting gender (*n* = 463), 229 (49.5%) identified as men and 234 (50.5%) as women; no respondents identified as nonbinary. Among all G2 partnerships, 29% were married or in registered partnerships, and 3.8% were non‐heterosexual. The mean age of G3 (45.5% girls) was 2.01 (*SD* = 0.25). The mean age of G1 mothers was 45.66 (*SD* = 4.20; range = 31–60 years) and of G1 fathers was 47.97 (*SD* = 4.95; range = 33–67 years) at T1. Among G1, 48.6% of mothers and 55.0% of fathers obtained a university degree at T1, which was higher than the proportion in the Dutch population in the same period (30.1%; OECD, 2005). The visual representation of ages for G1, G2, and G3 throughout the study is presented in Figure 1.

### Measures

#### Aggressive conflict resolution

G1 and G2 aggressive conflict resolution toward intimate partners at T1 and T2 was assessed using self‐reports on the Conflict Engagement subscale of the Dutch adapted version of the Conflict Resolution Style Inventory (CRSI; Kurdek, 1994). This subscale consisted of 5 items measuring the frequency of using specific aggressive measures to resolve disputes between intimate partners (e.g., “Exploding and getting out of control”). Items were rated on a 5‐point Likert scale ranging from 1 (*never*) to 5 (*always*). Mean scores were calculated, with higher scores indicating greater use of aggressive conflict resolution. The CRSI has evidence for construct, concurrent, and predictive validity (Kurdek, 1994) and can be used to assess conflict management behaviors in different types of relationships (Trifan et al., 2024). In this study, the measures showed acceptable to good internal consistency, Cronbach's 𝛼 = .79 (G1 father), = .79 (G1 mother), = .82 (G2 participant), and = .78 (G2 partner).

#### Harsh discipline

G2 harsh discipline toward G3 at T3 was assessed using self‐reports on the Harsh Discipline subscale of the Comprehensive Early Childhood Parenting Questionnaire (CECPAQ; Verhoeven et al., 2017). This subscale consisted of 12 items measuring the frequency with which parents employ physical, verbal, or psychological aggressive punishment toward their children (e.g., “I spank my child for whining”). Items were rated on a 6‐point Likert scale ranging from 1 (*never*) to 6 (*always*), except for the item “When my child misbehaves…,” which had response options ranging from “I raise my voice/yell” to “I speak to my child calmly” and was reversely coded. Mean scores were calculated, with higher scores indicating greater use of harsh discipline. Reliability and criterion validity of the CECPAQ have been supported (Verhoeven et al., 2017). In this study, the measure showed acceptable internal consistency (Cronbach's 𝛼 = .75).

#### Inconsistent discipline

G2 inconsistent discipline toward G3 at T3 was assessed using self‐reports on the Parental Dimensions Inventory (PDI; Deković et al., 2003; Slater & Power, 1987). The PDI consisted of 8 items measuring the extent to which parents exhibit inconsistent parenting practices over time toward their children (e.g., “Sometimes it takes so long for me to get a chance to respond to my child's violation that I just let it go”). Items were rated on a 6‐point Likert scale ranging from 1 (*strongly disagree*) to 6 (*strongly agree*). Mean scores were calculated, with higher scores indicating greater use of inconsistent discipline. Reliability and validity of the PDI were established in a previous study (Slater & Power, 1987). In the current study, the measure showed acceptable internal consistency (Cronbach's 𝛼 = .69).

All questionnaire items are presented in the Supplement S2. We tested whether harsh and inconsistent discipline could be combined into a single measure by conducting confirmatory factor analyses (CFA). If the results suggested an acceptable or good fit of the one‐factor model, a composite variable would be constructed to represent this combined measure. Results suggested that the two‐factor model, with items loading separately onto harsh and inconsistent discipline, showed acceptable fit, whereas the one‐factor model did not. Therefore, we conducted separate main analyses treating harsh and inconsistent discipline as distinct dependent variables. Details of the CFA results are presented in the Supplement S3. Model fit indices and comparisons of one‐factor and two‐factor models are presented in the Supplement S3 (Table S1).

### Data analytic strategy

Data preparation and descriptive analyses were conducted in R version 4.3.1 (R Core Team, 2023). Little's Missing Completely at Random (MCAR) test (Little, 1988) was used to determine whether the missing pattern was at random. Outliers were identified if scores deviated three standard deviations from the mean. In sensitivity analyses, we tested whether results with winsorized outliers differed from those with non‐winsorized data.

Main analyses were conducted using path analyses in M*plus* 8.8 (Muthén & Muthén, 2017) with the R MplusAutomation package (Hallquist & Wiley, 2018). As some participants were siblings, the interdependencies between siblings were accounted for by using TYPE = COMPLEX in M*plus* to adjust for standard errors and model test statistics with robust standard errors. Missing data were addressed using Full Information Maximum Likelihood (FIML) estimation. We used Maximum Likelihood of Robust Standard Errors (MLR) estimation to address nonnormality.

Two separate models for G1 father and G1 mother were specified. The models tested both the intergenerational transmission and spillover hypotheses, controlling for potential confounds by including G2's age at T2 as a covariate. The intergenerational transmission hypothesis was tested with regression paths from G1's aggressive conflict resolution at T1 to G2's and G2 partner's aggressive conflict resolution at T2. The spillover hypothesis was tested with regression paths from G2's and G2 partner's aggressive conflict resolution at T2 to G2's harsh and inconsistent discipline at T3. We also tested whether the indirect effect from G1's aggressive conflict resolution to G2's harsh and inconsistent discipline was significant. G2's and G2 partner's aggressive conflict resolution at T2 were allowed to correlate. Additionally, the model included a regression path from G1's aggressive conflict resolution at T1 to G2's harsh and inconsistent discipline at T3. Last, multiple group comparisons were performed to investigate the moderating effects of G2 gender on the regression paths of intergenerational transmission and spillover, by comparing each regression path across gender using Wald tests.

The model fit was assessed using recommended fit index cutoff values (Schreiber et al., 2006). Acceptable models were identified by the following criteria: the Comparative Fit Index (CFI) > .90 and Root Mean Square Error of Approximation (RMSEA) and Standardized Root Mean Square Residual (SRMR) < .08. Good model fits were indicated by CFI > .95 and RMSEA and SRMR < .05. Modification Indices (MIs) were considered to improve fit if the initial fit was unacceptable. Models in main analyses were compared using the Satorra‐Bentler Scaled Chi‐Squared Difference Test (∆S‐Bꭓ^2^; Satorra & Bentler, 2010). The significance level was .05. Correlation values of .10, .30, and .50 represented small, medium, and large effects, respectively (Cohen, 1992).

Sensitivity analyses were performed to compare the results in the full sample (*N* = 1178), which comprised all families, and the triad‐only sample, which comprised families only when G3 were involved (*n* = 222). If sensitivity analyses showed the same significance patterns and (nearly) identical parameter estimates between the two samples, the full sample would be used to present the findings considering the greater power it provided.

### Transparency and openness

We reported the determination of sample size, data exclusions, and all measures used in the study. The analysis code and research materials are available at https://osf.io/xn7u5. The research questions, hypotheses, and analysis plan have been preregistered on the Open Science Framework (https://osf.io/xn7u5).

## RESULTS

### Preliminary analyses

#### Descriptives

The descriptive statistics and zero‐order correlations of the variables are presented in the Supplement S4 (Table S2). Overall, the study variables (i.e., G1 and G2 aggressive conflict resolution and G2 harsh and inconsistent discipline) had a right‐skewed distribution. As expected, correlations of aggressive conflict resolution between G1 and G2 and between G2 and G2's partner were significant. Except for the correlation between G2 partner's aggressive conflict resolution and G2's inconsistent discipline, all correlations between G2 (partner)'s aggressive conflict resolution and G2's discipline were significant.

#### Attrition and missing data

The Little's MCAR test indicated that data were missing completely at random (*p* = .295). T‐tests and chi‐squared tests were performed to compare study variables between the full sample and triad‐only sample. Results suggested that for G2, the triad‐only sample consisted of a higher proportion of girls/women (63.6% for triad‐only sample; 51.5% for full sample), ꭓ^2^(1) = 11.12 (*p* < .001), and older participants (*M*_*triad‐only*_ = 15.09; *M*_*full*_ = 14.82) at T1, *t*(272.49) = −3.72 (*p* < .001).

### Main analyses

#### Structural path and mediation analysis

**FIGURE 2:**
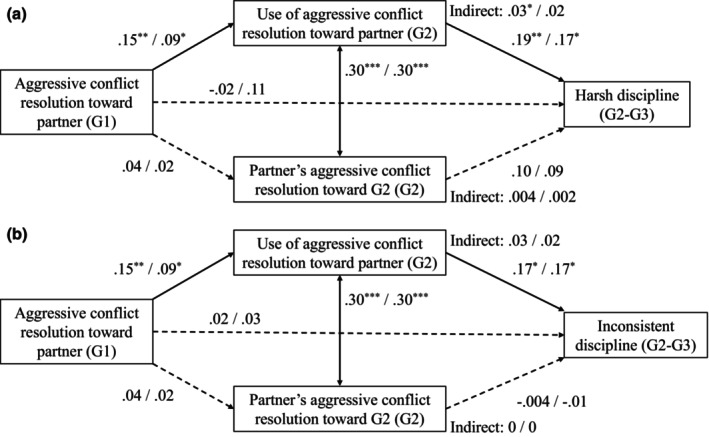
Path models with standardized parameter estimates. (A) Model predicting harsh discipline. (B) Model predicting inconsistent discipline. Dashed lines indicate insignificant paths. Values on the left side of slashes are based on G1 mother model (G1 mother's aggressive conflict resolution as G1 measure), and the right side based on G1 father model (G1 father's aggressive conflict resolution as G1 measure). *p < .05, **p < .01, ***p < .001.

The models, including all hypothesized paths and covariates, were saturated. Tables 1 and 2 present the (un)standardized parameter estimates, standard errors, and 95% confidence intervals. Figure 2a,b displays a visual representation of the main results. The sensitivity analysis showed that between the full sample and triad‐only sample, the significance patterns were the same and the parameter estimates were largely identical, with differences in ranging from .01 to .10. Therefore, the findings are presented based on the full sample. The results of the triad‐only sample are presented in the Supplement S5 (Table S3).

**TABLE 1:** Parameter estimates in path models predicting harsh discipline.

Parameter	G1 mother model^a^			G1 father model^b^		
	*B* (SE)	β	95% CI	*B* (SE)	β	95% CI
G1 ACR → G2 ACR	0.14 (0.04)**	.15	[0.06, 0.23]	0.10 (0.04)*	.09	[0.01, 0.18]
G1 ACR → G2 partner ACR	0.04 (0.04)	.04	[−0.04, 0.12]	0.02 (0.05)	.02	[−0.07, 0.11]
G2 ACR → G2 HD	0.11 (0.04)**	.19	[0.03, 0.19]	0.10 (0.04)*	.17	[0.02, 0.18]
G2 partner ACR → G2 HD	0.06 (0.05)	.10	[−0.04, 0.16]	0.06 (0.05)	.09	[−0.05, 0.16]
G1 ACR → G2 HD	−0.01 (0.05)	−.02	[−0.10, 0.08]	0.07 (0.04)	.11	[−0.01, 0.14]
G2 ACR ← → G2 partner ACR	0.10 (0.02)***	.30	[0.07, 0.13]	0.10 (0.02)***	.30	[0.07, 0.14]
G1 ACR → G2 ACR → G2 HD	0.02 (0.01)*	.03	[0.001, 0.03]	0.01 (0.01)	.02	[−0.002, 0.02]
G1 ACR → G2 partner ACR → G2 HD	0.002 (0.003)	.004	[−0.004, 0.01]	0.001 (0.003)	.002	[−0.01, 0.01]
Control paths						
G2 age at T2 → G2 ACR	−0.01 (0.01)	−.03	[−0.03, 0.01]	−0.01 (0.01)	−.04	[−0.03, 0.01]
G2 age at T2 → G2 partner ACR	−0.01 (0.01)	−.04	[−0.04, 0.01]	−0.01 (0.01)	−.04	[−0.04, 0.01]
G2 age at T2 → G2 HD	−0.03 (0.01)***	−.13	[−0.04, −0.01]	−0.03 (0.01)***	−.13	[−0.04, −0.01]

Abbreviations: ACR, aggressive conflict resolution; CI, confidence interval; HD, Harsh discipline.

^a^ G1 mother models: mother's aggressive conflict resolution as a predictor.

^b^ G1 father models: father's aggressive conflict resolution as a predictor.

* *p* < .05.

** *p* < .01.

*** *p* < .001.

**TABLE 2:** Parameter estimates in path models predicting inconsistent discipline.

Parameter	G1 mother model^a^			G1 father model^b^		
	*B* (*SE*)	β	95% CI	*B* (*SE*)	β	95% CI
G1 ACR → G2 ACR	0.15 (0.04)**	.15	[0.06, 0.23]	0.10 (0.04)*	.09	[0.01, 0.18]
G1 ACR → G2 partner ACR	0.04 (0.04)	.04	[−0.04, 0.12]	0.02 (0.05)	.02	[−0.07, 0.12]
G2 ACR → G2 ID	0.21 (0.09)*	.17	[0.03, 0.38]	0.20 (0.08)*	.17	[0.04, 0.37]
G2 partner ACR → G2 ID	−0.01 (0.10)	−.004	[−0.19, 0.18]	−0.01 (0.09)	−.01	[−0.19, 0.18]
G1 ACR → G2 ID	0.02 (0.09)	.02	[−0.16, 0.20]	0.04 (0.09)	.03	[−0.13, 0.21]
G2 ACR ←→ G2 partner ACR	0.10 (0.02)***	.30	[0.07, 0.14]	0.10 (0.02)***	.30	[0.07, 0.14]
G1 ACR → G2 ACR → G2 ID	0.03 (0.02)	.03	[−0.001, 0.06]	0.02 (0.01)	.02	[−0.003, 0.04]
G1 ACR → G2 partner ACR → G2 ID	0 (0.004)	0	[−0.01, 0.01]	0 (0.002)	0	[−0.004, 0.004]
Control paths						
G2 age at T2 → G2 ACR	−0.01 (0.01)	−.04	[−0.03, 0.01]	−0.01 (0.01)	−.04	[−0.03, 0.01]
G2 age at T2 → G2 partner ACR	−0.01 (0.01)	−.04	[−0.04, 0.01]	−0.01 (0.01)	−.04	[−0.04, 0.01]
G2 age at T2 → G2 ID	0.03 (0.02)*	.08	[0.001, 0.07]	0.03 (0.02)*	.08	[0.001, 0.07]

Abbreviations: ACR, aggressive conflict resolution; CI, confidence interval; ID, inconsistent discipline.

^a^ G1 mother models: mother's aggressive conflict resolution as a predictor.

^b^ G1 father models: father's aggressive conflict resolution as a predictor.

* *p* < .05.

** *p* < .01.

*** *p* < .001.

Regarding the intergenerational transmission hypothesis, G1 mothers' and fathers' aggressive conflict resolution at T1 was significantly and positively associated with G2's aggressive conflict resolution at T2. These associations were small for both G1 mothers and fathers. The associations between G1 mother's and father's aggressive conflict resolution and G2 partner's aggressive conflict resolution were not significant. Additionally, moderate, significant and positive within‐time correlations of aggressive conflict resolution between G2 and G2's partner at T2 were found in all models.

Concerning the spillover hypothesis, G2's aggressive conflict resolution at T2 was significantly and positively, but weakly, related to G2's harsh and inconsistent discipline at T3. The relations between G2 partner's aggressive conflict resolution and G2's harsh and inconsistent discipline were not significant.

No significant direct associations were found between G1's aggressive conflict resolution at T1 and G2's harsh and inconsistent discipline at T3. However, mediation analyses indicated significant and small indirect relations from G1 mothers' aggressive conflict resolution to G2's harsh discipline via G2's aggressive conflict resolution. However, indirect relations from G1 mother's aggressive conflict resolution to G2's inconsistent discipline and from G1 father's aggressive conflict resolution to G2's harsh and inconsistent discipline were not significant. Also, G2 partner's aggressive conflict resolution was not a significant mediator in the relations between G1's aggressive conflict resolution and G2's harsh and inconsistent discipline.

Regarding the control paths, G2's age was not associated with either G2's or G2 partner's aggressive conflict resolution. However, G2's age was negatively related to G2's harsh discipline and positively related to G2's inconsistent discipline.

A combined model including both G1 parents' aggressive conflict resolution was also tested, yielding results broadly consistent with the separate models. Two effects became nonsignificant: G1 fathers' effects on G2's aggressive conflict resolution and G2 mothers' indirect effects on G2's harsh discipline via G2's conflict resolution. Results from the combined model can be found in the Supplement S6 (Table S4).

#### Multiple group analysis

Multiple group analyses were conducted to explore potential G2 gender differences in the intergenerational transmission of aggressive conflict resolution from G1 to G2 (partner) and spillover relations from G2 (partner)'s aggressive conflict resolution to G2's harsh and inconsistent discipline. The models with specified gender groups were saturated. Table 3 presents the multiple group analysis results and parameter estimates in gender groups.

**TABLE 3:** Wald tests and standardized parameters for multiple group analyses.

Model	G1 mother model^a^					G1 father model^b^				
	Men β	Women β	Wald χ^2^	*Df*	*p*	Men β	Women β	Wald χ^2^	*Df*	*p*
Model predicting harsh discipline										
G1 ACR → G2 ACR	.12	.16**	0.95	1	.330	.14	.03	1.10	1	.296
G1 ACR → G2 partner ACR	.05	.02	0.10	1	.756	.08	−.02	1.13	1	.288
G2 ACR → G2 HD	.10	.26**	0.64	1	.426	.08	.25**	0.63	1	.420
G2 partner ACR → G2 HD	.16	.06	0.24	1	.625	.14	.05	0.22	1	.637
Model predicting inconsistent discipline										
G1 ACR → G2 ACR	.12	.16**	0.96	1	.328	.14	.04	1.01	1	.314
G1 ACR → G2 partner ACR	.06	.02	0.11	1	.737	.08	−.02	1.16	1	.281
G2 ACR → G2 ID	.08	.29**	1.24	1	.265	.002	.31**	4.05	1	.044
G2 partner ACR → G2 ID	.12	−.12	2.67	1	.102	.08	−.12	2.11	1	.147

*Note*: Men = standardized parameters in the G2 men group; Women = standardized parameters in the G2 women group.

Abbreviations: ACR, aggressive conflict resolution; HD, Harsh discipline; ID, Inconsistent discipline.

^a^ G1 mother models: mother's aggressive conflict resolution as a predictor.

^b^ G1 father models: father's aggressive conflict resolution as a predictor.

** *p* < .01.

Wald tests revealed nonsignificant differences between gender groups for all paths, except for the spillover relation from G2's aggressive conflict resolution with their partner to G2's inconsistent discipline when G1 father was treated as the predictor. Specifically, when G1 father was the exogenous variable, compared to G2 women, the relation from G2 men's conflict resolution with their partner to their inconsistent discipline was significantly stronger.

### Sensitivity analysis for outliers

Sensitivity analyses were performed to assess the impact of outliers on the results. Outliers in aggressive conflict resolution of G1 mother (*n* = 7) and father (*n* = 7), G2 (*n* = 10) and G2's partner (*n* = 3), and G2 harsh discipline (*n* = 2) were winsorized. Between the winsorized data and the nonwinsorized data, there were no differences in significance patterns and only minor changes in the standardized parameters, typically around .01. This suggested that outliers did not substantially influence the results. The parameter estimates based on the winsorized sample are provided in the Supplement S7 (Table S5).

## DISCUSSION

The current study used a prospective longitudinal design to examine the intergenerational transmission of aggressive conflict resolution toward intimate partners, the potential spillover effects from aggressive conflict resolution to later harsh and inconsistent discipline, and the adolescent gendered moderation on the transmission and spillover relations. Our results revealed that parents' aggressive conflict resolution in adolescents' mid‐adolescence was related to adolescents' aggressive conflict resolution later in adulthood. However, parents' aggressive conflict resolution was not related to adolescents' partner's aggressive conflict resolution. In addition, adolescents' aggressive conflict resolution was associated with their later use of harsh and inconsistent discipline, while this spillover relation was not found for their partner's aggressive conflict resolution. Additionally, adolescents' and their partner's aggressive conflict resolution were found to be associated. Last, there was an indirect relation between mothers' aggressive conflict resolution and adolescents' harsh discipline through adolescents' aggressive conflict resolution.

### Intergenerational transmission of aggressive conflict resolution

Our findings indicated that adolescents whose parents employed more aggressive conflict resolution strategies were more likely to use similar strategies in their own intimate relationships, supporting our hypothesis grounded in the Social Learning Theory (Bandura, 1977; Ehrensaft & Langhinrichsen‐Rohling, 2022; Kerr & Capaldi, 2019). This finding aligned with prior evidence on the intergenerational transmission of family conflict intensity (Hsieh et al., 2022; Rothenberg et al., 2016) and relational aggression (Cui et al., 2010; Doumas et al., 1994), but extended these findings by focusing specifically on aggression within conflict contexts. Adolescents raised in families where aggressive conflict resolution is prevalent may internalize these behaviors and reproduce them in their romantic relationships in young adulthood. This finding could inform early interventions supporting parents in adopting constructive conflict management strategies before their children reach adolescence, aiming to disrupt cycles of aggressive conflict resolution.

Contrary to our expectations based on Social Learning Theory and partner selection effects (Cui et al., 2010; Bandura, 1977), we did not find a significant association between parents' aggressive conflict resolution during adolescence and child partners' conflict behaviors during young adulthood. This null finding suggests that the continuity of family conflicts identified in earlier studies (Hsieh et al., 2022; Rothenberg et al., 2016) may primarily manifest through direct parent‐to‐child transmission, rather than through adolescents' selection into similarly aggressive relationships.

Nevertheless, the positive concurrent association between participants' and their partners' aggressive conflict resolution suggests behavior contagion effects within couples (Rhule‐Louie & McMahon, 2007) and reciprocal behavior in conflict interactions. Prior research indicates that partners mutually influence one another, resulting in behavioral concordance over time (Ehrensaft & Langhinrichsen‐Rohling, 2022; Rhule‐Louie & McMahon, 2007). This raises the possibility of indirect intergenerational transmission in which adolescents' own aggressive conflict resolution may serve as a pathway through which parental conflict resolution was indirectly associated with their partners' behavior. However, longitudinal studies with repeated, temporally ordered measures of parental conflict resolution behaviors as well as both participants' and their partners' conflict resolution behaviors are necessary to test such mediation and reciprocal influence more rigorously.

### Spillover effects of aggressive conflict resolution to harsh and inconsistent discipline

Our findings revealed participants who used aggressive conflict resolution with their partners were more likely to discipline their children harshly and inconsistently later. This supported our hypothesis and is consistent with theoretical frameworks (Bandura, 1973) and prior empirical evidence (Buehler & Gerard, 2002; Kaczynski et al., 2006; McCoy et al., 2013; Sears et al., 2016; Van Dijk et al., 2020; Warmuth et al., 2020). This suggests that individuals who rely on aggression to resolve disputes with partners may similarly view coercive, aggressive, and contrasting discipline as effective strategies for managing child behavior (Bandura, 1973).

Contrary to our expectations, we did not find evidence that participants' partners' use of aggressive conflict resolution predicted participants' harsh and inconsistent discipline. This null finding diverged from previous research suggesting that exposure to partners' aggression may deplete parents' emotional resources and impair their capacity for effective and consistent discipline (Kaczynski et al., 2006; Van Dijk et al., 2020; Warmuth et al., 2020). One possible explanation lies in the nature of our sample and measurement. Prior studies often examined severe partner aggression or used high‐risk samples (e.g., Sypher et al., 2022), whereas our study focused on a community sample and assessed a relatively common and less extreme form of aggressive conflict resolution. Thus, the less intense and more normative nature of the conflict behaviors in our study may have yielded weaker associations with parenting outcomes.

Notably, partners' aggressive conflict resolution was significantly associated with adolescents' harsh discipline prior to accounting for adolescents' own aggressive conflict resolution. However, once adolescents' conflict resolution was included in the model, the partners' contribution was no longer significant. This pattern may reflect the reciprocal influence between adolescents and their partners in conflict resolution, as mentioned (Rhule‐Louie & McMahon, 2007), suggesting that the partners' behavior may indirectly affect parenting through its association with the adolescents' own behavior. Given that adolescents' conflict resolution had a stronger direct effect on parenting, the partners' influence may be partially mediated or masked by this variable. Future research with repeated, temporally ordered assessments of both adolescents' and partners' conflict resolution behaviors is needed to clarify potential bi‐directional processes and their implications for parenting.

### Direct effects of parents' aggressive conflict resolution on adolescents' discipline

Contrary to our hypothesis, we did not find a significant direct association between parents' aggressive conflict resolution and adolescents' subsequent discipline. This finding contrasted with prior research indicating that exposure to inter‐parental aggression can directly predict later negative parenting (Lotto et al., 2023; Rowell & Neal‐Barnett, 2022). One possible explanation is that the way parents resolve disputes with each other may not directly shape adolescents' future parenting. Instead, other relational dynamics in the family of origin—such as more recent conflict interactions between parents or parent–child interactions during adolescence—may have a more direct and stronger influence. Indeed, studies suggested that aggression directed towards the child, as opposed to aggression between parents, may exert a stronger impact on children's later psychosocial adjustment and relational behavior (Cui et al., 2010). It is therefore possible that parent‐to‐child conflict resolution, rather than interparental conflict alone, may be a more proximal predictor of adolescents' parenting practices. Future studies could include measures of parent–child conflict resolution to test its potential direct effects on the next generation's parenting.

### Role of parent and adolescent gender

#### Intergenerational transmission and spillover effects

Our findings revealed no significant differences by gender of either parent or adolescent in the intergenerational transmission of aggressive conflict resolution, consistent with previous research on aggression and antisocial behavior (Cui et al., 2010; Kalmuss, 1984; Kwong et al., 2003; Li et al., 2023; Smith et al., 2011; Tzoumakis et al., 2020). In other words, the transmission was equally robust across men and women in both generations. This finding is important for informing the design of interventions aimed at breaking the cycle of aggressive conflict behaviors: efforts may need to target both parental figures and both adolescent sons and daughters either in adolescence or young adulthood, regardless of specific gender configurations. A combined model with both G1 parents in the sensitivity analysis showed the G2 fathers' effects on G2's aggressive conflict resolution became nonsignificant. Given that fathers' effects remained significant when tested separately, this attenuation may reflect overlapping variance between mothers' and fathers' conflict behaviors, with mothers' effects capturing shared variance when modeled simultaneously.

We further extended the literature by examining adolescent gender as a potential moderator of spillover effects from adolescents' romantic conflict to their discipline—an area that remains understudied (Kopystynska et al., 2017). Overall, the gendered moderation was largely nonsignificant, except for a stronger spillover from adolescent women's conflict resolution to their inconsistent discipline when predicted by their father's conflict behavior. This finding resonated with prior research suggesting that women with high levels of aggression may experience greater difficulty adjusting to caregiving roles (Elder et al., 1986; Rothenberg et al., 2016). However, this effect should be interpreted with caution, as it was not replicated in models where the mother was the predictor, and the statistical significance of the interaction was marginal. Although our hypothesis – guided by Social Role Theory (Thompson & Walker, 1989) and prior findings (e.g., Levenson & Gottman, 1983; McCoy et al., 2013; Stroud et al., 2011)—anticipated stronger spillover effects for men, we found no consistent evidence for moderation by adolescent gender across the majority of our models. One possible explanation is that previous studies examined overall dyadic functioning (e.g., Stroud et al., 2011), whereas we disaggregated individual and partner conflict behaviors. Our results suggested that when focusing on individual‐level, instead of dyadic‐level, conflict behaviors, gender differences in spillover found in prior research were not observed.

Importantly, the consistent spillover effect from participants' aggressive conflict resolution to their parenting – regardless of gender – suggested that it was the use of aggressive conflict behavior, rather than exposure to it, that was most predictive of later harsh and inconsistent discipline. This underscores the importance of targeting the “actor” in conflict resolution, whether men or women, in future interventions aimed at promoting healthier parenting practices.

#### Mediation of aggressive conflict resolution used by adolescents and partners

Although we did not observe direct relations between parental aggressive conflict resolution and adolescents' discipline, we found that mother's aggressive conflict resolution was indirectly related to adolescents' use of harsh discipline through adolescents' aggressive conflict resolution. This supported our hypothesis and was consistent with prior two‐ and three‐generational studies suggesting both the intergenerational transmission of aggression and its spillover into parenting behaviors (e.g., Conger et al., 2003; Neppl et al., 2019). The mediating role of participants' conflict resolution highlights the importance of young adults' conflict management strategies: adolescents appeared to learn from and reproduce the aggressive interaction patterns modeled by their parents in their own adulthood, first in their own romantic relationships and subsequently in their parenting practices. This finding suggested that targeting conflict resolution skills during adolescence and young adulthood may be promising for interventions aimed at disrupting the intergenerational cycle of aggressive conflict behaviors within families.

In contrast to mothers, fathers' aggressive conflict resolution did not show a significant indirect effect. One possible explanation for this gender difference lies in traditional caregiving roles: mothers often assume primary caregiving responsibilities and typically spend more time with their children than fathers, which may amplify their influence on children's interpersonal behaviors (Craig & Mullan, 2011). Also, contrary to our expectations, indirect effects on adolescents' inconsistent discipline were not significant. One possible explanation is that harsh discipline may share more behavioral and affective similarities with aggressive conflict resolution—such as coerciveness or emotional reactivity—compared to inconsistent discipline, which may reflect more disorganization or lack of structure. Therefore, adolescents may be more likely to replicate aggressive conflict patterns through harsh rather than inconsistent disciplinary practices.

### Strengths, limitations, and future directions

The current study has several strengths. First, it used a longitudinal and prospective study design across a span of 15 years. According to intergenerational transmission reviews (Branje et al., 2020; Kerr & Capaldi, 2019), much research has risked potential recall bias for using cross‐sectional approaches to test longitudinal relations. Hence, our findings were more robust in testing long‐term associations that require variables tested in a temporal order (Thornberry et al., 2012). Second, the inclusion of both fathers and mothers in the first generation enabled us to incorporate both parental figures to test the associations between G1's and G2's behavior (Branje et al., 2020). This inclusion further allowed us to differentiate and compare the transmission of conflict resolution across the four parent–child dyads based on gender. Third, our three‐generation study is one of the first to link intergenerational transmission and spillover effects together. Compared with two‐generation studies, our findings provide a more expansive view in understanding the relation between interparental conflict resolution and parental discipline across three generations. It is also through this three‐generation perspective that prevention efforts could recognize the need to support adolescents while anticipating their future parenting role (Kerr & Capaldi, 2019).

Despite these strengths, several limitations warrant consideration. First, the reliance on self‐reports may introduce reporter bias. Second, variability in the transmission may arise from the differing ages at which conflict resolution was assessed for the first and second generations. Because behaviors may evolve across the life course, individuals' conflict strategies might reflect different traits at different developmental stages. Future studies could examine intergenerational transmission within similar life phases to better capture behavioral continuity (Branje et al., 2020; Thornberry, 2016). Similarly, parenting may differ across children's developmental stages (Barry & Kochanska, 2010); for example, harsh and inconsistent parenting may vary in form and intensity at different ages. Comparing processes of intergenerational transmission in parenting behaviors at different child ages may help identify patterns of consistency and change across generations. Third, this study did not allow for a clear disentanglement of all potential mechanisms underlying the intergenerational transmission. While social learning may be one plausible explanation, alternative pathways, including genetic (D'Onofrio et al., 2007), psychological (e.g., emotional dysregulation; Kim, Pears, et al., 2009), and social processes (e.g., parenting; Cui et al., 2010) may also contribute. Future studies using genetically informed designs or longitudinal mediation models can help disentangle these mechanisms and offer a more comprehensive understanding of the processes that sustain conflict behaviors across generations.

## CONCLUSION

Using a longitudinal sample spanning multiple generations, the current study showed that parents' harsh and inconsistent discipline is related to their aggressive conflict resolution style in intimate partnerships, which can be traced back to their own parents' aggressive conflict resolution. Moreover, the intergenerational transmission and spillover relations remained largely consistent for both parent and adolescent gender. The present study underscores the importance of targeting both partners of the first and second generations to interrupt the intergenerational transmission of aggressive conflict resolution, mitigate problematic disciplinary strategies, and foster healthier family dynamics across generations.

## AUTHOR CONTRIBUTIONS

**Pin Chen:** Conceptualization; data curation; formal analysis; writing – original draft; writing – review and editing; visualization. **Susan Branje:** Writing – review and editing; supervision. **Sanne B. Geeraerts:** Conceptualization; writing – review and editing; supervision.

## FUNDING INFORMATION

Data of the RADAR (Research on Adolescent Development And Relationships) study were used (https://doi.org/10.17026/dans‐zrb‐v5wp). RADAR was founded by W. Meeus and has been financially supported by main grants from the Netherlands Organization for Scientific Research (GB‐MAGW 480‐03‐005, GB‐MAGW 480‐08‐006) and Stichting Achmea Slachtoffer en Samenleving (SASS) and a grant from the European Research Council (ERC‐2017‐CoG‐773,023 INTRANSITION). The funders had no role in the study design, data collection and analysis, decision to publish, or preparation of the manuscript.

## CONFLICT OF INTEREST STATEMENT

The authors have no conflict of interest to disclose.

## ETHICS STATEMENT

Ethical approval was initially obtained from the University of Medical Centre Utrecht and for later waves by the Ethics Review Board of the Faculty of Social and Behavioral Sciences of Utrecht University (FETC24‐0157) on January 13, 2024.

## PATIENT CONSENT STATEMENT

All participants and their parents/guardians provided active consent.

## Supporting information


Data S1:

## Data Availability

The data that support the findings of this study are available upon request from the corresponding author. The data are not publicly available due to privacy or ethical restrictions.
