# Supplementary material for: Learning from the past: Intergenerational transmission of aggressive conflict resolution between intimate partners predicts harsh and inconsistent parenting
Source: J Res Adolesc. 2025 Nov 30;35(4):e70102. doi: 10.1111/jora.70102 (PMC12665178; doi:10.1111/jora.70102)
Supplement: Supplementary file 1 — Data S1: [file JORA-35-0-s001.docx]

**Supplementary Materials**

**Supplement 1: Data Collection and Participant Inclusion**

***Data Collection***

The current study used data from the ongoing Dutch longitudinal study, The Research on Adolescent Development and Relationships (RADAR; Branje & Meeus, 2018; Van Lier et al., 2011). Ethical approval was initially obtained from *xxx (anonymized)* and for later waves by *xxx (anonymized)*. Two cohorts of participants, the RADAR Old and RADAR Young, from randomly selected high schools in the Netherlands began participating in their first year of high school in 2001 and 2005, respectively. In addition to target adolescents (Generation 2, G2), RADAR also includes assessments of adolescents’ parents (Generation 1, G1), siblings, best friends, partners, and their siblings’ partners. G2 participants (both target adolescents and siblings) were invited for data collection in Generation 3 (RADAR-G3) once they had children (G3).

The current study used data from both cohorts of participants, their parents, intimate partners, and children when the children were two years old. This also means that some participants may come from the same family. All participants provided active consent. Retention rates (85%) are very high (Branje et al. 2020). The data collection procedure has been well documented by Branje et al. (2020) and Van Lier et al. (2011).

***Participant Inclusion***

The current study focused on the inter-parental use of aggressive conflict resolution during participants’ mid-adolescence and its associations with: (1) adolescents’ own use of aggressive conflict resolution, (2) adolescents’ partners’ use of aggressive conflict resolution, and (3) adolescents’ subsequent disciplinary practices toward their two-year-old children. For this purpose, three time points were defined:

1. T1: The wave during which G2 participants were in mid-adolescence (ages 14-17),
2. T2: The last wave in which G2 participated prior to the assessment of their parenting, and
3. T3: The wave in which G3 children were two years old.

To assess inter-parental conflict resolution at T1, we extracted the waves when the target G2 participants were 14 years old and obtained reports of both G1 parents. To maximize the sample size, siblings of the target G2 participants were also included if they were within the 14–17-year age range (i.e., middle adolescence) during the same wave, which introduced some age variability among adolescent participants within each wave.

To examine spillover effects, it was necessary to ensure that adolescents’ and their partners’ reported levels of aggressive conflict resolution were measured at a time point (T2) that closely proceeded T3. Thus, we first identified T3 by extracting the wave when each G3 child was two years old, and subsequently defined T2 as the preceding wave for each G2 participant.

To further maximize data utilization, we included T2 data of G2 participants who did not have children and therefore lacked data at T3. For these participants, we calculated the modal interval of waves between T1 and T2 in the dataset of participants with T3 data (i.e., eight waves) and used this interval to assign a T2 wave for participants without children participating. These participants, by design, had available data only at T1 and T2.

The final analytical sample consisted of 1,178 G1-G2 dyads (including 443 pairs of G2 siblings) and 222 G1-G2-G3 triads (including 28 pairs of G2 siblings).

**Supplement 2: Questionnaire Overview**

***Aggressive Conflict Resolution***

Measure: Conflict Resolution Engagement - Conflict Resolution Style Inventory (CRSI)

Source: Kurdek (1994)

1. Personally attack him/her
2. Exploding and getting out of control
3. Getting carried away and saying things that aren’t meant
4. Throwing insults and digs
5. Getting so angry that I do not know what I am doing anymore

***Harsh Discipline***

Measure: Comprehensive Early Childhood Parenting Questionnaire (CECPAQ)

Source: Verhoeven et al. (2017)

1. When my child misbehaves...I raise my voice, or yell/I speak to my child calmly
2. When my child disobeys, I get angry and raise my voice
3. When my child is whining, I get angry and raise my voice
4. I slap my child when s/he has done something wrong
5. When my child misbehaves, I get angry, and grab hold of him/her
6. I spank my child for whining
7. When my child does something I don’t like, I insult my child
8. I tell my child that s/he should be ashamed when s/he misbehaves
9. When my child does something I don’t like, I scowl at him/her and pretend he/she does not exist
10. I make my child feel guilty when s/he doesn’t meet my expectations
11. When my child does something I don’t like, I don’t talk to him/her until he/she behaves better
12. I criticize my child when s/he doesn’t meet my expectations

***Inconsistent Discipline***

Measure: Parental Dimension Inventory (PDI)

Source: Deković et al. (2003); Slater & Power (1987)

1. Sometimes it takes so long for me to get a chance to respond to my child's violation that I just let it go
2. Sometimes I just don't have the energy to get my child to behave as he or she should
3. My child often manages to persuade me to punish him/her more lightly than I intended
4. My child convinces me to change my mind after I reject his/her request
5. I have little or no difficulty enforcing rules on my child, even when family (including grandparents) are present
6. Once I have decided how to deal with my child's bad behavior, I stick to it
7. I threaten punishment only when I am sure I will carry it out
8. I always persevere in disciplining my child, no matter how long it takes

**Supplement 3: Confirmatory Factor Analysis**

Table S1 presents the model fit indices and fit improvement for the one-factor and two-factor confirmatory factor analysis models of G2 harsh and inconsistent discipline. Initially, the one-factor model with all items loading onto a single latent factor demonstrated poor fit. Following the addition of 13 correlation paths between items suggested by Modification Indices with overlapping wording (e.g., “*I tell my child that s/he should be ashamed when s/he misbehaves*” and “*I make my child feel guilty when s/he doesn’t meet my expectations*”), the model significantly improved. However, the model fit remained inadequate, leading us to test the two-factor model with harsh and inconsistent discipline specified as two distinct latent variables. The two-factor model also suggested poor fit initially. Therefore, 11 correlation paths between items suggested by Modification Indices with overlapping wording were added. This resulted in an adjusted two-factor model with significant improvement in the fit. Since the adjusted two-factor model showed acceptable fit, we conducted separate main analyses with harsh and inconsistent discipline as distinct dependent variables.

**Table S1**

*Model Fit Indices and Comparison for Confirmatory Factor Analyses*

| Model | ꭓ^2^(*df*) | *p* | CFI | RMSEA [90% CI] | SRMR | S-Bꭓ^2^(*df*) | *p* for S-Bꭓ^2^ |
| --- | --- | --- | --- | --- | --- | --- | --- |
| One-factor model ^a^ |  |  |  |  |  | 264.59(13) | < .001 |
| Initial model | 829.036(170) | < .001 | .339 | .135 [.126, .145] | .122 |  |  |
| Adjusted model | 291.235(157) | < .001 | .865 | .064 [.052, .075] | .074 |  |  |
| Two-factor model ^b^ |  |  |  |  |  | 215.54(11) | < .001 |
| Initial model | 675.336(169) | < .001 | .492 | .119 [.110, .128] | .104 |  |  |
| Adjusted model | 249.445 (158) | < .001 | .910 | .052 [.040, .064] | .069 |  |  |

*Note.* Initial model is the model before adding suggested paths by Modification Indices. Adjusted model is the model with additional correlation paths between item variables suggested by Modification Indices. Values for S-Bꭓ^2^(*df*) and *p* for S-Bꭓ^2^ are results of model comparison between initial models and adjusted models.

^a^ One-factor model is the model with harsh and inconsistent discipline specified as a single latent factor. ^b^ Two-factor model is the model with harsh and inconsistent discipline specified as two distinct latent factors.

**Supplement 4**

**Table S2**

*Means, Standard Deviations, and Zero-Order Correlations for Study Variables*

| Variable | *n* | *M* (*SD*) | Range | 1 | 2 | 3 | 4 | 5 | 6 | 7 | 8 |
| --- | --- | --- | --- | --- | --- | --- | --- | --- | --- | --- | --- |
| 1. G2 gender ^a^ | 1,135 | 0.51 (0.50) | 0-1 | – |  |  |  |  |  |  |  |
| 2. G2 age (T2) | 1,066 | 26.92 (1.69) | 18.35-34.21 | -.05 | – |  |  |  |  |  |  |
| 3. G1 mother ACR (T1) | 1,118 | 1.81 (0.61) | 1.00-4.20 | .03 | -.01 | – |  |  |  |  |  |
| 4. G1 father ACR (T1) | 1,104 | 1.61 (0.54) | 1.00-4.80 | .05 | .01 | .25^***^ | – |  |  |  |  |
| 5. G2 ACR (T2) | 618 | 1.55 (0.59) | 1.00-5.00 | .22^**^ | -.04 | .15^***^ | .10^*^ | – |  |  |  |
| 6. G2 partner ACR (T2) | 532 | 1.71 (0.58) | 1.00-3.80 | -.09^*^ | -.06 | .05 | .03 | .28^***^ | – |  |  |
| 7. G2 Harsh Discipline (T3) | 212 | 1.62 (0.35) | 1.00-3.20 | .06 | -.23^***^ | .06 | .15^*^ | .23^***^ | .17^*^ | – |  |
| 8. G2 Inconsistent Discipline (T3) | 212 | 2.30 (0.69) | 1.00-4.10 | -.11 | .12 | .05 | .05 | .17^*^ | .05 | .18^**^ | – |

*Note.* ACR = Aggressive Conflict Resolution.

^a^ G2 gender was measured as a dichotomous variable (0 = men, 1 = women).

^*^*p* < .05, ^**^*p* < .01, ^***^*p* < .001.

**Supplement 5**

**Table S3**

*Parameter Estimates in Path Models with Triad-Only Sample*

| Parameter | *B* (*SE*) | 𝛽 | 95% CI |
| --- | --- | --- | --- |
| G1 mother model ^a^ |  |  |  |
| Model predicting harsh discipline |  |  |  |
| G1 ACR 🡪 G2 ACR | 0.25 (0.07)^**^ | .25 | [0.11, 0.39] |
| G1 ACR 🡪 G2 partner ACR | 0.12 (0.08) | .12 | [-0.03, 0.27] |
| G2 ACR 🡪 G2 HD | 0.11 (0.04)^**^ | .19 | [0.03, 0.19] |
| G2 partner ACR 🡪 G2 HD | 0.06 (0.05) | .10 | [-0.04, 0.16] |
| G1 ACR 🡪 G2 HD | -0.02 (0.05) | -.03 | [-0.11, 0.07] |
| G2 ACR 🡨🡪 G2 partner ACR | 0.09 (0.02)^***^ | .27 | [0.002, 0.13] |
| G1 ACR 🡪 G2 ACR 🡪 G2 HD | 0.03 (0.01)^*^ | .05 | [0.002, 0.05] |
| G1 ACR 🡪 G2 partner ACR 🡪 G2 HD | 0.01 (0.01) | .01 | [-0.01, 0.02] |
| Control paths |  |  |  |
| G2 age at T2 🡪 G2 ACR | -0.02 (0.01) | -.09 | [-0.04, 0.01] |
| G2 age at T2 🡪 G2 partner ACR | -0.01 (0.02) | -.05 | [-0.04, 0.02] |
| G2 age at T2 🡪 G2 HD | -0.03 (0.01)^***^ | -.22 | [-0.04, -0.01] |
| Model predicting inconsistent discipline |  |  |  |
| G1 ACR 🡪 G2 ACR | 0.26 (0.07)^***^ | .25 | [0.11, 0.40] |
| G1 ACR 🡪 G2 partner ACR | 0.12 (0.08) | .13 | [-0.04, 0.27] |
| G2 ACR 🡪 G2 ID | 0.21 (0.09)^*^ | .18 | [0.04, 0.38] |
| G2 partner ACR 🡪 G2 ID | -0.004 (0.09) | -.003 | [-0.19, 0.18] |
| G1 ACR 🡪 G2 ID | 0.02 (0.09) | .02 | [-0.16, 0.20] |
| G2 ACR 🡨🡪 G2 partner ACR | 0.09 (0.02)^***^ | .27 | [0.04, 0.13] |
| G1 ACR 🡪 G2 ACR 🡪 G2 ID | 0.05 (0.03) | .05 | [-0.003, 0.11] |
| G1 ACR 🡪 G2 partner ACR 🡪 G2 ID | 0 (0.01) | 0 | [-0.02, 0.02] |
| Control paths |  |  |  |
| G2 age at T2 🡪 G2 ACR | -0.02 (0.01) | -.09 | [-0.04, 0.01] |
| G2 age at T2 🡪 G2 partner ACR | -0.01 (0.02) | -.06 | [-0.04, 0.02] |
| G2 age at T2 🡪 G2 ID | 0.03 (0.12)^***^ | .14 | [0.002, 0.06] |
| G1 father model ^b^ |  |  |  |
| Model predicting harsh discipline |  |  |  |
| G1 ACR 🡪 G2 ACR | 0.17 (0.08)^*^ | .15 | [0, 0.33] |
| G1 ACR 🡪 G2 partner ACR | 0.13 (0.09) | .12 | [-0.04, 0.30] |
| G2 ACR 🡪 G2 HD | 0.10 (0.04)^*^ | .17 | [0.02, 0.18] |
| G2 partner ACR 🡪 G2 HD | 0.05 (.05) | .09 | [-0.05, 0.15] |
| G1 ACR 🡪 G2 HD | 0.06 (0.04) | .10 | [-0.01, 0.14] |
| G2 ACR 🡨🡪 G2 partner ACR | 0.09 (0.02)^***^ | .28 | [0.04, 0.13] |
| G1 ACR 🡪 G2 ACR 🡪 G2 HD | 0.02 (0.01) | .03 | [-0.003, 0.04] |
| G1 ACR 🡪 G2 partner ACR 🡪 G2 HD | 0.01 (0.01) | .01 | [-0.01, 0.02] |
| Control paths |  |  |  |
| G2 age at T2 🡪 G2 ACR | -0.02 (0.01) | -.09 | [-0.05, 0.01] |
| G2 age at T2 🡪 G2 partner ACR | -0.01 (0.02) | -.06 | [-0.04, 0.02] |
| G2 age at T2 🡪 G2 HD | -0.03 (0.01)^***^ | -.22 | [-0.04, -0.01] |
| Model predicting inconsistent discipline |  |  |  |
| G1 ACR 🡪 G2 ACR | 0.17 (0.08)^*^ | .15 | [0.01, 0.34] |
| G1 ACR 🡪 G2 partner ACR | 0.14 (0.09) | .13 | [-0.04, 0.31] |
| G2 ACR 🡪 G2 ID | 0.21 (0.08)^*^ | .18 | [0.04, 0.37] |
| G2 partner ACR 🡪 G2 ID | -0.01 (0.09) | -.004 | [-0.19, 0.18] |
| G1 ACR 🡪 G2 ID | 0.04 (0.09) | .03 | [-0.13, 0.21] |
| G2 ACR 🡨🡪 G2 partner ACR | 0.09 (0.02)^***^ | .28 | [0.04, 0.13] |
| G1 ACR 🡪 G2 ACR 🡪 G2 ID | 0.04 (0.02) | .03 | [-0.01, 0.08] |
| G1 ACR 🡪 G2 partner ACR 🡪 G2 ID | -0.001 (0.01) | -.001 | [-0.03, 0.02] |
| Control paths |  |  |  |
| G2 age at T2 🡪 G2 ACR | -0.02 (0.01) | -.10 | [-0.05, 0.01] |
| G2 age at T2 🡪 G2 partner ACR | -0.01 (0.02) | -.06 | [-0.04, 0.02] |
| G2 age at T2 🡪 G2 ID | 0.03 (0.02)^*^ | .14 | [0.002, 0.06] |

*Note.* CI = Confidence Interval; ACR = Aggressive Conflict Resolution; HD = Harsh Discipline; ID = Inconsistent Discipline.

^a^ G1 mother models are path models with G1 mother’s aggressive conflict resolution as the exogenous variable. ^b^ G1 father models are path models with G1 father’s aggressive conflict resolution as the exogenous variable.

^*^*p* < .05, ^**^*p* < .01, ^***^*p* < .001.

**Supplement 6**

**Table S4**

*Parameter Estimates in Path Models with Both G1 Measures*

| Parameter | *B* (*SE*) | 𝛽 | 95% CI |
| --- | --- | --- | --- |
| Model predicting harsh discipline |  |  |  |
| G1 Mother ACR 🡪 G2 ACR | 0.13 (0.05)^**^ | .14 | [0.04, 0.22] |
| **G1 Father ACR 🡪 G2 ACR** | **0.06 (0.05)** | **.05** | **[-0.03, 0.15]** |
| G1 Mother ACR 🡪 G2 partner ACR | 0.04 (0.04) | .04 | [-0.05, 0.12] |
| G1 Father ACR 🡪 G2 partner ACR | 0.01 (0.05) | .01 | [-0.09, 0.10] |
| G2 ACR 🡪 G2 HD | 0.11 (0.04)^*^ | .18 | [0.02, 0.19] |
| G2 partner ACR 🡪 G2 HD | 0.06 (0.05) | .10 | [-0.05, 0.16] |
| G1 Mother ACR 🡪 G2 HD | -0.03 (0.05) | -.06 | [-0.12, 0.06] |
| G1 Father ACR 🡪 G2 HD | 0.07 (0.04) | .12 | [-0.01, 0.16] |
| G2 ACR 🡨🡪 G2 partner ACR | 0.10 (0.02)^***^ | .30 | [0.07, 0.13] |
| G1 Mother ACR 🡨🡪 G1 Father ACR | 0.08 (0.01)^***^ | .25 | [0.06, 0.11] |
| **G1 Mother ACR 🡪 G2 ACR 🡪 G2 HD** | **0.01 (0.01)** | **.03** | **[-0.001, 0.03]** |
| G1 Father ACR 🡪 G2 ACR 🡪 G2 HD | 0.01 (0.01) | .01 | [-0.004, 0.02] |
| G1 Mother ACR 🡪 G2 partner ACR 🡪 G2 HD | 0.002 (0.003) | .004 | [-0.004, 0.01] |
| G1 Father ACR 🡪 G2 partner ACR 🡪 G2 HD | 0 (0.003) | .001 | [-0.01, 0.01] |
| Control paths |  |  |  |
| G2 age at T2 🡪 G2 ACR | -0.01 (0.01) | -.03 | [-0.03, 0.01] |
| G2 age at T2 🡪 G2 partner ACR | -0.01 (0.01) | -.04 | [-0.04, 0.01] |
| G2 age at T2 🡪 G2 HD | -0.03 (0.01)^***^ | -.13 | [-0.04, -0.01] |
| Model predicting inconsistent discipline |  |  |  |
| G1 Mother ACR 🡪 G2 ACR | 0.13 (0.05)^**^ | .14 | [0.04, 0.22] |
| **G1 Father ACR 🡪 G2 ACR** | **0.06 (0.05)** | **.06** | **[-0.03, 0.15]** |
| G1 Mother ACR 🡪 G2 partner ACR | 0.04 (0.04) | .04 | [-0.05, 0.12] |
| G1 Father ACR 🡪 G2 partner ACR | 0.01 (0.05) | .01 | [-0.09, 0.11] |
| G2 ACR 🡪 G2 ID | 0.20 (0.09)^*^ | .17 | [0.03, 0.38] |
| G2 partner ACR 🡪 G2 ID | -0.01 (0.09) | -0.01 | [-0.19, 0.18] |
| G1 Mother ACR 🡪 G2 ID | 0.01 (0.09) | .01 | [-0.16, 0.19] |
| G1 Father ACR 🡪 G2 ID | 0.04 (0.09) | .03 | [-0.13, 0.21] |
| G2 ACR 🡨🡪 G2 partner ACR | 0.10 (0.02)^***^ | .30 | [0.07, 0.14] |
| G1 Mother ACR 🡨🡪 G1 Father ACR | 0.08 (0.01)^***^ | .25 | [0.06, 0.11] |
| G1 Mother ACR 🡪 G2 ACR 🡪 G2 ID | 0.03 (0.02) | .02 | [-0.004, 0.06] |
| G1 Father ACR 🡪 G2 ACR 🡪 G2 ID | 0.01 (0.01) | .01 | [-0.01, 0.03] |
| G1 Mother ACR 🡪 G2 partner ACR 🡪 G2 ID | 0 (0.003) | 0 | [-0.01, 0.01] |
| G1 Father ACR 🡪 G2 partner ACR 🡪 G2 ID | 0 (0.001) | 0 | [-0.002, 0.002] |
| Control paths |  |  |  |
| G2 age at T2 🡪 G2 ACR | -0.01 (0.01) | -.04 | [-0.03, 0.01] |
| G2 age at T2 🡪 G2 partner ACR | -0.01 (0.01) | -.04 | [-0.04, 0.01] |
| G2 age at T2 🡪 G2 ID | 0.03 (0.02)^*^ | .08 | [0.001, 0.06] |

*Note.* CI = Confidence Interval; ACR = Aggressive Conflict Resolution; HD = Harsh Discipline; ID = Inconsistent Discipline. Bolded rows indicate results that differ from the main models (i.e., separate G1 mother and father models in the main text).

^*^*p* < .05, ^**^*p* < .01, ^***^*p* < .001.

**Supplement 7**

**Table S5**

*Parameter Estimates in Path Models with Winsorized Sample*

| Parameter | *B* (*SE*) | 𝛽 | 95% CI |
| --- | --- | --- | --- |
| G1 mother model ^a^ |  |  |  |
| Model predicting harsh discipline |  |  |  |
| G1 ACR 🡪 G2 ACR | 0.14 (0.04)^***^ | .16 | [0.06, 0.22] |
| G1 ACR 🡪 G2 partner ACR | 0.04 (0.04) | .04 | [-0.04, 0.12] |
| G2 ACR 🡪 G2 HD | 0.12 (0.04)^**^ | .19 | [0.03, 0.20] |
| G2 partner ACR 🡪 G2 HD | 0.05 (0.05) | .08 | [-0.04, 0.14] |
| G1 ACR 🡪 G2 HD | -0.01 (0.05) | -.02 | [-0.10, 0.08] |
| G2 ACR 🡨🡪 G2 partner ACR | 0.09 (0.02)^***^ | .29 | [0.06, 0.12] |
| G1 ACR 🡪 G2 ACR 🡪 G2 HD | 0.02 (0.01)^*^ | .03 | [0.002, 0.03] |
| G1 ACR 🡪 G2 partner ACR 🡪 G2 HD | 0.002 (0.003) | .003 | [-0.004, 0.01] |
| Control paths |  |  |  |
| G2 age at T2 🡪 G2 ACR | -0.01 (0.01) | -.04 | [-0.03, 0.01] |
| G2 age at T2 🡪 G2 partner ACR | -0.01 (0.01) | -.04 | [-0.04, 0.01] |
| G2 age at T2 🡪 G2 HD | -0.03 (0.01)^***^ | -.13 | [-0.04, -0.01] |
| Model predicting inconsistent discipline |  |  |  |
| G1 ACR 🡪 G2 ACR | 0.15 (0.04)^***^ | .16 | [0.07, 0.23] |
| G1 ACR 🡪 G2 partner ACR | 0.04 (0.04) | .04 | [-0.04, 0.12] |
| G2 ACR 🡪 G2 ID | 0.21 (0.09)^*^ | .17 | [0.03, 0.39] |
| G2 partner ACR 🡪 G2 ID | -0.01 (0.10) | -.004 | [-0.19, 0.18] |
| G1 ACR 🡪 G2 ID | 0.02 (0.09) | .02 | [-0.16, 0.20] |
| G2 ACR 🡨🡪 G2 partner ACR | 0.09 (0.02)^***^ | .29 | [0.06, 0.12] |
| G1 ACR 🡪 G2 ACR 🡪 G2 ID | 0.03 (0.01) | .03 | [-0.002, 0.06] |
| G1 ACR 🡪 G2 partner ACR 🡪 G2 ID | 0 (0.004) | 0 | [-0.01, 0.01] |
| Control paths |  |  |  |
| G2 age at T2 🡪 G2 ACR | -0.01 (0.01) | -.04 | [-0.03, 0.01] |
| G2 age at T2 🡪 G2 partner ACR | -0.01 (0.01) | -.04 | [-0.04, 0.01] |
| G2 age at T2 🡪 G2 ID | 0.03 (0.02)^*^ | .08 | [0.001, 0.06] |
| G1 father model ^b^ |  |  |  |
| Model predicting harsh discipline |  |  |  |
| G1 ACR 🡪 G2 ACR | 0.10 (0.04)^*^ | .10 | [0.02, 0.19] |
| G1 ACR 🡪 G2 partner ACR | 0.02 (0.05) | .02 | [-0.07, 0.12] |
| G2 ACR 🡪 G2 HD | 0.11 (0.04)^*^ | .18 | [0.02, 0.19] |
| G2 partner ACR 🡪 G2 HD | 0.04 (0.05) | .07 | [-0.05, 0.13] |
| G1 ACR 🡪 G2 HD | 0.06 (0.04) | .10 | [-0.011, 0.14] |
| G2 ACR 🡨🡪 G2 partner ACR | 0.09 (0.02)^***^ | .29 | [0.06, 0.12] |
| G1 ACR 🡪 G2 ACR 🡪 G2 HD | 0.01 (0.01)^*^ | .02 | [-0.001, 0.02] |
| G1 ACR 🡪 G2 partner ACR 🡪 G2 HD | 0.001 (0.002) | .002 | [-0.004, 0.01] |
| Control paths |  |  |  |
| G2 age at T2 🡪 G2 ACR | -0.02 (0.01) | -.04 | [-0.03, 0.01] |
| G2 age at T2 🡪 G2 partner ACR | -0.01 (0.01) | -.04 | [-0.04, 0.01] |
| G2 age at T2 🡪 G2 HD | -0.03 (0.01)^***^ | -.13 | [-0.04, -0.01] |
| Model predicting inconsistent discipline |  |  |  |
| G1 ACR 🡪 G2 ACR | 0.10 (0.04)^*^ | .10 | [0.02, 0.19] |
| G1 ACR 🡪 G2 partner ACR | 0.03 (0.05) | .02 | [-0.07, 0.12] |
| G2 ACR 🡪 G2 ID | 0.21 (0.09)^*^ | .17 | [0.04, 0.38] |
| G2 partner ACR 🡪 G2 ID | -0.01 (0.10) | -.01 | [-0.19, 0.17] |
| G1 ACR 🡪 G2 ID | 0.04 (0.09) | .03 | [-0.13, 0.22] |
| G2 ACR 🡨🡪 G2 partner ACR | 0.09 (0.02)^***^ | .29 | [0.06, 0.12] |
| G1 ACR 🡪 G2 ACR 🡪 G2 ID | 0.02 (0.01) | .02 | [-0.003, 0.05] |
| G1 ACR 🡪 G2 partner ACR 🡪 G2 ID | 0 (0.002) | 0 | [-0.01, 0.01] |
| Control paths |  |  |  |
| G2 age at T2 🡪 G2 ACR | -0.01 (0.01) | -.04 | [-0.03, 0.01] |
| G2 age at T2 🡪 G2 partner ACR | -0.01 (0.01) | -.04 | [-0.04, 0.01] |
| G2 age at T2 🡪 G2 ID | 0.03 (0.02)^*^ | .08 | [0.002, 0.06] |

*Note.* CI = Confidence Interval; ACR = Aggressive Conflict Resolution; HD = Harsh Discipline; ID = Inconsistent Discipline.

^a^ G1 mother models are path models with G1 mother’s aggressive conflict resolution as the exogenous variable. ^b^ G1 father models are path models with G1 father’s aggressive conflict resolution as the exogenous variable.

^*^*p* < .05, ^**^*p* < .01, ^***^*p* < .001.

**Supplement 8**

**Figure S1**

*Eligibility and Participant Inclusion*


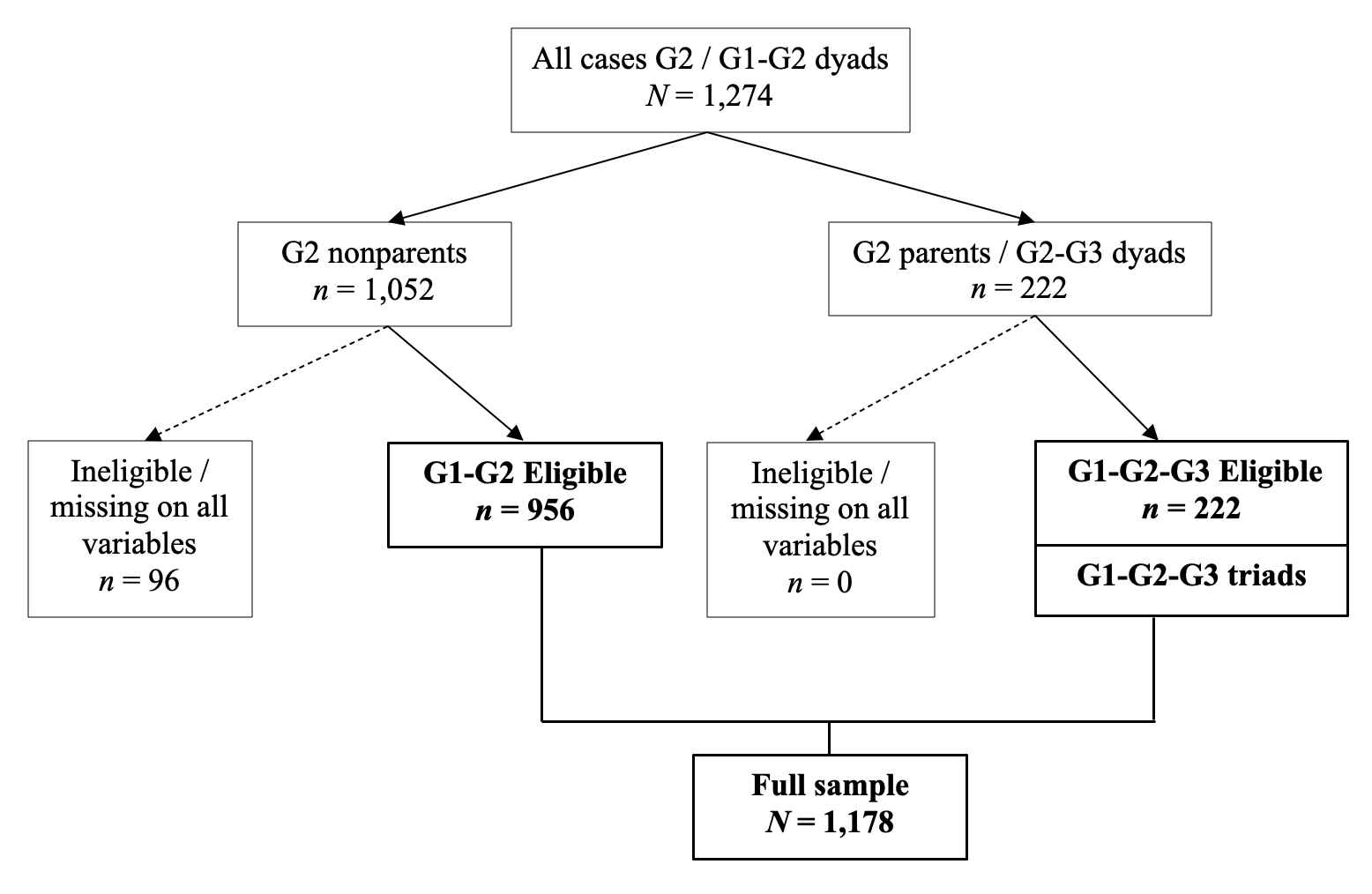


*Note.* Based on Supplement 1, all waves were extracted and participants were screened for inclusion. Participants with missing data on all variables of interest (*n* = 96) were excluded from the current study.
